# Olive Pomace and Soybean-Sunflower Acid Oils as Alternative Fat Sources in European Seabass (*Dicentrarchus labrax*) Diets: Effects on Performance, Digestibility and Flesh Fatty Acid Composition and Quality Parameters

**DOI:** 10.3390/ani12091198

**Published:** 2022-05-06

**Authors:** Gerard Verge-Mèrida, Ana Cristina Barroeta, Carlos Ferrer, Tània Serrano, Francesc Guardiola, María Dolores Soler, Roser Sala

**Affiliations:** 1 Animal Nutrition and Welfare Service (SNiBA), Department of Animal and Food Sciences, Universitat Autònoma de Barcelona, 08193 Bellaterra, Spain; gerard.verge@uab.cat (G.V.-M.); ana.barroeta@uab.cat (A.C.B.); carlos.ferrer@e-campus.uab.cat (C.F.); tania.serrano@e-campus.uab.cat (T.S.); 2 Nutrition, Food Science and Gastronomy Department, Institut de Recerca en Nutrició i Seguretat Alimentària, Xarxa d’Innovació Alimentària, Universitat de Barcelona, 08921 Santa Coloma de Gramenet, Spain; fguardiola@ub.edu; 3 Department of Animal Production and Health, Universidad CEU Cardenal Herrera, 46115 Valencia, Spain; mariola@uchceu.es

**Keywords:** acid oil, free fatty acid, fat by-product, alternative energy source, dietary fat, flesh quality, fish nutrition

## Abstract

**Simple Summary:**

Acid oils, by-products of edible oil refining, are potentially interesting fat sources for farmed fish diets because of their high energy content and usually competitive price. Their use and revaluation may contribute to more efficient and sustainable fish production. They are characterised by presenting a similar fatty acid profile to their respective crude oils, but with a high content of free fatty acids. The present study aimed to investigate the effects of including soybean-sunflower and olive pomace acid oils in European seabass diets, as a preliminary step to determine whether they might be suitable energy sources for fish diets. The results showed that growth was only impaired in animals fed the diet containing olive pomace acid oil, which had the highest moisture, impurities and unsaponifiable matter. They also suggest that dietary free fatty acid content affects digestibility, but not the fatty acid profile of flesh and perivisceral fat. Notwithstanding, further studies assessing the effects of the inclusion of these oils are needed before recommending their use.

**Abstract:**

The effects of dietary inclusion of soybean-sunflower and olive pomace acid oils on growth, digestibility and flesh composition were studied in European seabass. Eight diets were fed for 100 days (101.37 ± 0.33 g initial weight, mean ± SD), differing in the added fat source (25% fish oil, 75% experimental oil): S (crude soybean oil), SA (soybean-sunflower acid oil), O (crude olive pomace oil) or OA (olive pomace acid oil); 3 blends: S-O, S-OA, SA-OA at a 1:1 ratio; and a diet containing only fish oil (F) as a control. Animals fed OA showed the worst performance among dietary treatments, with the lowest weight, specific growth ratio, average daily gain and the highest feed conversion ratio (*p* < 0.01). In contrast, other diets including acid oils did not impair performance. Acid oil diets did not affect the apparent digestibility of dry matter, crude protein or total fatty acids (*p* > 0.05), but a lower digestibility of lipids and saturated fatty acids was observed (*p* < 0.001). Flesh composition and fatty acid profile were not affected by the high dietary free FA content (*p* > 0.05). Hence the results suggest that the studied acid oils may potentially be used in fish diets although further studies are needed.

## 1. Introduction

The increasing importance of aquaculture, linked to factors such as population growth, the increasing demand for aquatic food products and the bioaccumulation of toxic compounds in wild marine species, has raised the need for safe and efficient production of aquatic species. In Mediterranean aquaculture, European seabass (*Dicentrarchus labrax*) is one of the most important farmed marine fish species, Turkey, Greece, Spain and Egypt being the countries that account for 88% of total production [1].

In farmed fish diets, fish oil (FO) had traditionally been used as the only dietary fat source, partly due to its energy content but mainly to its contribution of eicosapentaenoic (EPA; C20:5 n − 3) and docosahexaenoic (DHA; C22:6 n − 3) acids. These n − 3 highly unsaturated fatty acids (n − 3 HUFA) are considered essential for marine fish [2]. However, since the global supply is insufficient to cover the increasing demand for FO in aquaculture, the need arose to find sustainable alternative oil sources, and major research efforts in recent years have studied different strategies and alternatives for the replacement of FO with vegetable oils (VO) in fish diets [3,4]. Aquaculture production is expected not only to be efficient but also sustainable, so circularity should be one of the cornerstones of future aquaculture feeds [5]. Results of studies carried out in broiler chickens and pigs suggest that soybean-sunflower and olive pomace acid oils are by-products of edible oil refining that can be used as potential alternative fat sources [6,7,8]. These acid oils are obtained by chemically refining crude oils, which consists of many steps, including degumming, bleaching, deodorization and alkali neutralization. Essentially, the refining process of crude oils consists of removing free FA (FFA) and other non-desirable compounds in order to obtain a refined oil suitable for human consumption, and acid oils are generated as by-products [9]. Hence, acid oils are characterised by a similar fatty acid (FA) profile to their respective crude oils, but with a higher content of free FA (FFA) (40–60%; [8,10]). They are also cost-effective as they are usually competitively priced, and are readily available to fish feed manufacturers at the European level since soybean and sunflower oils are two of the most produced VO worldwide and the extraction of olive pomace oil is mainly concentrated in the Mediterranean arc [11]. In fact, soybean-sunflower acid oil is the most widely available in the European market. Information about the effects of oils rich in FFA on farmed fish species is scarce, and only a few studies using palm fatty acid distillate (90% of FFA) or rapeseed acid oil (47% of FFA) in rainbow trout (*Oncorhynchus mykiss*) [12,13,14,15] and gilthead seabream (*Sparus aurata*) [16] have been found in the literature.

The potential use of a new ingredient requires the assessment of its quality and composition. According to Glencross [17], the characterization of ingredients is a critical step in the evaluation process. In this sense, the characterization of soybean-sunflower and olive pomace acid oils has been reported by [10,18]. Therefore, the objective of the present study was to investigate the effects of including soybean-sunflower and olive pomace acid oils in European seabass diets on growth performance, digestibility and flesh composition, as a preliminary step to determining whether they might be suitable energy sources for fish diets.

## 2. Materials and Methods

### 2.1. Experimental Fats and Diets

Eight experimental diets were formulated to be isoproteic and isolipidic using the same ingredient composition except for the added fat source (15.4% of the diet, as-fed basis). The added fat consisted of 25% FO and 75% experimental oil. Then, four diets including experimental oils, namely S (crude soybean oil diet), SA (soybean-sunflower acid oil diet), O (crude olive pomace oil diet) and OA (olive pomace acid oil diet); and three blends at a 1:1 ratio (diet S-O; diet S-OA; diet SA-OA) were formulated. A diet was formulated including only commercial fish oil for use as a control (F). Diets were formulated according to the nutritional requirements of the species [19]. Ingredients and proximate composition of the experimental diets are shown in Table 1. Yttrium oxide (Y_2_O_3_) was added to the diets as an inert marker for digestibility balance.

Crude soybean oil and soybean-sunflower acid oil (approximately 55:45, *w*/*w*) were supplied by Bunge Ibérica S.A.U. (Sant Just Desvern, Spain). Crude olive pomace oil was supplied by General d’Olis i Derivats S.L. (Borges Blanques, Spain) and olive pomace acid oil was supplied by RIOSA S.A. (Refinación Industrial Oleícola S.A., Ibros, Spain). Comercial FO was obtained from AFAMSA (Agrupación de Fabricantes de Aceites Marinos, S.A., Mos, Spain). The experimental diets were manufactured as extruded pellets by Ceimar-University of Almeria (Experimental Diets Service, Almeria, Spain) using standard aquafeed procedures. Briefly, feed ingredients were finely ground and mixed in a vertical helix ribbon mixer (Sammic BM−10, 10-L capacity, Sammic, Azpeitia, Spain) before oil and diluted choline chloride were added. All the ingredients were mixed together for 20 min, and then water (350 mL/kg) was added to the mixture to obtain a homogeneous dough. The dough was passed through a single screw laboratory extruder (Miltenz 51SP, JSConwell Ltd., New Zealand). The extruder barrel consisted of four sections, and the temperature profile in each section (from inlet to outlet) was 95 °C, 98 °C, 100 °C, and 110 °C, respectively. Finally, pellets were dried at 27 °C in a drying chamber (Airfrio, Almeria, Spain) for 24 h and feeds were kept in sealed plastic bags at −20 °C until use.

### 2.2. Fish Husbandry and Sampling

All the procedures were conducted following the European Union Guidelines for the ethical care and handling of animals under experimental conditions (2010/63/EU) and in accordance with the Animal Protocol Review Committee of the Universitat Autònoma de Barcelona (CEEAH). The trial was carried out at the Aquaculture Center facilities of the Institute of Agrifood Research and Technology (IRTA, Sant Carles de la Ràpita, Spain). A total of 480 European seabass (with an average of 101.37 ± 0.33 g body weight, mean ± SD) were randomly allocated into 24 cylindroconical tanks with a capacity of 500 L (20 fish per tank) in a sea water recirculation system (IRTAmar^®^; IRTA, Sant Carles de la Ràpita, Spain). This system allows for water recirculation of between 1 and 1.5 tank volumes per hour (15 m^3^/h), and is equipped with an aerobic biofilter for the removal/transformation of ammonia to nitrite and nitrite to nitrate. The supply of fresh water to the system consists of 5–15% of the total volume per day. Each experimental diet was randomly assigned to three tanks and was administered twice a day by automatic feeders (adjusted to provide 2–2.5% average BW daily; at 8.00 am and 2.00 pm) for 100 days. Uneaten feed was collected by filtering effluent water from each tank and collectors were emptied at the end of each meal, so the average feed intake per tank was recorded daily. Water temperature (22.55 ± 0.84 °C), dissolved oxygen levels (7.30 ± 0.66 mg/L), pH (7.9 ± 0.2) and salinity (35.5 ± 0.50‰) were maintained throughout the study. The levels of ammonia (0–0.5 ppm) and nitrites (0–2 ppm) were maintained within the safe levels for the species. During the experimental period (from July to October), the tanks were subjected to natural photoperiod.

All animals were weighed and measured individually at the beginning (day 0) and at the end of the experimental period (day 100). Each tank had a removable faecal settling system for the collection of faecal samples where feed and faeces are separated on the basis of their different densities. Faecal collection was carried out during the last two weeks of the experimental period and then stored at −20 °C until further analyses. At the end of the experimental period and after 24 h of fasting, six fish from each tank (18 animals per treatment) were euthanized by hypothermia in a mix of water and ice (1:3) and individually gutted. Viscera and abdominal fat pad were removed and weighed. The entire left and right muscles were also removed and weighed. The left muscle was immediately used for fresh colorimetric determination. The right muscle was cut into two different sections (dorsal and ventral, according to horizontal septum) and weighed. All samples were bagged individually and stored at −20 °C until chemical analyses.

### 2.3. Chemical Analyses

Prior to chemical analyses, samples of each experimental oil and feed were pooled, homogenized and kept at 5 °C. Faeces, skinned muscle (whole left muscle and the dorsal and ventral portions of the right muscle) and perivisceral fat samples were homogenized, freeze-dried (LyoAlpha 10/15; Telstar, Terrassa, Spain) and kept at 5 °C. Fatty acid composition, lipid class composition and MIU (moisture, impurities and unsaponifiable matter) content of experimental oils were analysed in duplicate as described by Varona et al. [18]. Analytical determinations for the chemical composition of the feeds, faeces and left muscle were performed according to AOAC International [20] methods: dry matter (934.01), ash (942.05), crude protein (954.01), ether extract by Soxhlet analysis (920.39) and crude fibre (962.09). The gross energy of feed and faeces was determined using an adiabatic bomb calorimeter (Parr 6300 Calorimeter, Parr Instrument Company, Moline, IL, USA) according to the UNE-EN ISO 9831:2004 standard. Liquid holding capacity analysis was carried out as described in Trullàs et al. [14]. Triplicate muscle samples of 3–4 cm were taken, weighed and placed in a tube with a weighed filter paper (Filter-Lab, Filtros Anoia, Sant Pere de Riudebitlles, Spain). Tubes were then placed in a centrifuge (Sigma 4K15, St. Louis, MO, USA) at 500 g for 10 min at 10 °C. Finally, the filter paper was dried at 50 °C until constant weight and drip, water and fat loss values were obtained. Liquid holding capacity assessment parameters were calculated as follows: water retained = (% total moisture − % water loss)/% total moisture; fat retained = (% total fat − % fat loss)/% total fat. Values were expressed as % of water and fat retained.

The FA content of feed and faeces were analysed following the method described by Sukhija and Palmquist [21]. Perivisceral fat and dorsal and ventral sections of the right muscle were analysed following the method described by Carrapiso et al. [22]. Nonadecanoic acid (C19:0; Sigma-Aldrich Chemical Co.; St. Louis, MO, USA) was added as an internal standard. The final extract obtained was injected in a gas chromatograph (HP6890, Agilent Technologies; Waldbronn, Germany) following the method and conditions described by Cortinas et al. [23].

### 2.4. Characterization of Experimental Oils and Diets

Lipid class composition and MIU values of experimental oils are shown in Table 2. Crude oils (FO, soybean oil, olive pomace oil) were mainly composed of triacylglycerols (TAG; >77%), while the main lipid class component in soybean-sunflower acid oil and olive pomace acid oil was FFAs (53.25% and 44.95%, respectively). Furthermore, acid oils showed higher MIU values than their respective crude oils, with olive pomace acid oil having the highest total MIU value (6.15%), due to its higher values for both impurities and unsaponifiable matter.

The FA composition of experimental oils and diets are shown in Table 2 and Table 3, respectively. Experimental diets showed a FA profile in accordance with the added experimental oils. Soybean diets (S and SA) were the richest in polyunsaturated fatty acids (PUFA; 53.61% and 48.62%, respectively) mainly due to their high linoleic acid content (C18:2 n − 6). In contrast, olive oil diets (O and OA) were the richest in monounsaturated fatty acids (MUFA; 52.84% and 48.22%, respectively), oleic acid (C18:1 n − 9) being the most abundant. Comparing acid with its corresponding crude oil diets, slightly higher MUFA and lower PUFA content were obtained for SA compared to S, while slightly lower MUFA and higher PUFA content were obtained for OA with respect to O. Diets with the experimental oil blends showed values close to the mean of those between the corresponding single oil diets. Among dietary treatments, the control diet (F) showed the highest percentage for saturated fatty acids (SFA; 33.11%), and n − 3:n − 6 ratio (2.09) due to having the highest EPA (6.98%) and DHA (23.21%) content.

### 2.5. Colour Evaluation of Flesh

Colorimetric determinations were performed on the fresh and thawed left muscle using a Minolta chroma meter (Model CR−410, Minolta Co., Osaka, Japan) on the Norwegian Quality Cut (NQC) section [24]. Thawed muscles were stored for six months at −20 °C and defrosted overnight at 4 °C the day prior to colorimetric assessment. Determinations were carried out in the colour space L*, a*, b* [25], where L* represents the lightness of the sample, a* defines the position between red/magenta and green and b* defines the position between yellow and blue. Then, C* (chroma, colour saturation) and h (hue angle) values were calculated as C* = (a*^2^ + b*^2^)^1/2^ and h = arctan (b*/a*), respectively [26]. Three measurements were performed on each of the six muscles per tank, and the mean value was used for statistical analysis.

### 2.6. Digestibility and Performance Parameter Calculations

All calculations were in accordance with standard formulae [27,28]. The apparent digestibility coefficient (ADC) of a particular nutrient or FA (X) was calculated as follows:

% ADC of X = {1 − [(Xf/Mf)/(Xd/Md)]} × 100,(1)

where Xf is the concentration of a particular nutrient or FA in faeces, Mf is the concentration of the inert marker in faeces, Xd is the concentration of a particular nutrient or FA in the diet, and Md is the concentration of the inert marker in the diet. The digestible energy of feeds was calculated from the product of energy ADC and its corresponding feed gross energy.

Growth performance and carcass parameters were calculated according to standard formulae. The average daily gain was calculated from:

ADG (g) = (final weight − initial weight)/numbers of days;(2)

average daily feed intake from:

ADFI = total feed intake (as-fed basis)/(number of fish ∗ number of days fed);(3)

feed conversion ratio from:

FCR = total feed fed (as-fed basis)/wet weight gain;(4)

specific growth rate from:

SGR = [(ln final weight − ln initial weight)/(number of days)] ∗ 100;(5)

condition factor from:

CF = (final weight/fork length)^3^ × 100; (6)

carcass yield from:

Carcass yield = [(body weight (BW) − visceral weight)/BW] ∗ 100;(7)

gross flesh yield from:

Gross flesh yield = (entire left and right muscle weight/BW) ∗ 100;(8)

net flesh yield from:

Net flesh yield = (entire left and right muscle weight/eviscerated carcass weight) ∗ 100;(9)

and perivisceral fat percentage from:

Perivisceral fat percentage = (perivisceral fat weight/BW) ∗ 100.(10)

### 2.7. Statistical Analysis

The normality of the data and homogeneity of variance were verified using the CAPABILITY procedure of SAS (version 9.4, SAS Institute Inc., Cary, NC, USA). All data were analysed using the GLM (general linear model) procedure of SAS. Differences between means were tested using Tukey’s adjust correction for multiple comparisons. For growth performance, digestibility balance, colorimetric and quality assessment of flesh and FA profile of muscle and perivisceral fat, the experimental unit was the tank. For carcass parameters, the experimental unit was the individual. The results in the tables are reported as the least square means, and differences were considered significant at *p* < 0.05.

## 3. Results

### 3.1. Performance and Carcass Parameters

The effects of added oils on growth performance and carcass parameters are shown in Table 4. Differences were obtained for all performance parameters among experimental diets throughout the experimental period, except for CF. At the end of the experimental period, animals fed OA showed the lowest BW (*p* = 0.002) ADG (*p* = 0.003) and SGR (*p* = 0.002) values among dietary treatments. Additionally, they had the highest FCR, which was significantly different to those fed F or SA (*p* = 0.004). In terms of ADFI, the lowest value was observed in animals fed F (*p* = 0.005).

Regarding carcass parameters, no differences among dietary treatments were observed in carcass weight or in the percentages of carcass yield, flesh yield and perivisceral fat (*p* > 0.05). Mean values of carcass yield and gross and net flesh yields were about 89%, 41% and 46%, respectively.

### 3.2. Digestibility Balance

Feed digestible energy and the ADC of macronutrients and FA are shown in Table 5. Experimental diets were well digested with an ADC for dry matter of about 96%. No differences were obtained for feed digestible energy or for the ADC of dry matter or crude protein (*p* > 0.05) among dietary treatments. In contrast, differences were observed in the ADC of lipids. Acid oil diets (SA, OA and SA-OA) showed a lower ADC for crude fat than their corresponding crude oil diets (S, O and S-O, respectively; *p* < 0.001). When comparing to F, no differences were observed for diets including crude oils (alone or in a blend), while lower values of crude fat ADC were obtained for diets composed only of acid oils (alone or in a blend) (*p* < 0.001).

Concerning ADC of FA, significant differences were observed among dietary treatments for total FA (TFA), SFA and MUFA, but not for PUFA, n − 3 or n − 6 FA. No significant differences were obtained for TFA and MUFA digestibility between diets containing acid oils and their corresponding crude oil (*p* > 0.05). For SFA, only SA showed lower digestibility than S (*p* < 0.05).

When acid oil diets (SA, OA and SA-OA) were compared to F, no significant differences in the ADC of FA were obtained (*p* > 0.05). In contrast, higher TFA digestibility was obtained for diets including crude soybean oil (S, S-O and S-OA) (*p* < 0.01). Similarly, diets with crude olive pomace oil (O and S-O) showed higher SFA digestibility than F (*p* < 0.001).

### 3.3. Flesh Composition and Quality Parameters

The colorimetric assessment, chemical composition and liquid holding capacity of the flesh are shown in Table 6. In fresh muscle, differences were only observed for the parameter L*, S-OA showing the lowest value among dietary treatments (*p* = 0.001). In thawed muscle, differences were observed for both C* and b* parameters, the flesh from diets O and S-O being those that showed the lowest values among dietary treatments (*p* < 0.01). When comparing fresh to thawed muscle, L* increased, while h and a* decreased (*p* < 0.001).

No differences were observed in the chemical composition of flesh. Regarding lipid content, no statistical differences were found among dietary treatments either for the dorsal or ventral sections. However, the ventral section of the muscle showed a higher lipid content than the dorsal (about 35% vs. 17% on average, respectively; *p* < 0.001). On the other hand, dietary treatments showed no differences in terms of the liquid holding capacity of thawed muscle.

### 3.4. Fatty Acid Profile of Flesh and Perivisceral Fat

The FA profiles of the dorsal and ventral sections of the muscle and perivisceral fat are presented in Table 7 and Table 8, respectively. In both tissues, differences observed in the FA profile among dietary treatments mirrored those of the FA profile of the experimental diets. Animals fed soybean oil diets (S or SA) had the highest PUFA (*p* < 0.001) content, while those fed olive pomace oil diets (O or OA) did for MUFA (*p* < 0.001). However, the differences in MUFA and PUFA composition observed between acid oil and their corresponding crude oil diets are more clearly reflected in the FA profile of perivisceral fat than in the two sections of muscle (dorsal and ventral). When compared with animals fed F, higher UFA: SFA and lower n − 3: n − 6 ratios (*p* < 0.001) were observed in animals fed VO diets. In perivisceral fat, the EPA and DHA contents in treatments including VO were about 30–33% and 43–52%, respectively, of those of F. Higher content (with respect to F) was obtained for the dorsal (56–62% for EPA; 45–52% for DHA) and ventral (54–60% for EPA; 42–57% for DHA). Moreover, higher DHA content was obtained for the dorsal muscle compared to the ventral muscle (*p* = 0.046), but no other significant differences were observed in the FA profile of the dorsal and ventral sections.

## 4. Discussion

### 4.1. Performance and Carcass Parameters

In the present study, the level of replacement of FO was formulated according to previous studies in rainbow trout and gilthead seabream [12,15,16,29] and to ensure the reported requirements of n − 3 HUFA for European seabass in older juvenile and pre-adult stages [19,30,31]. This level of replacement of FO (75%) with crude vegetable oils (soybean and olive pomace oils) did not affect the performance, achieving similar final weights and FCR. This is in agreement with other studies in European seabass, in which no differences in either SGR or feed utilisation efficiency were found with up to 60–80% of FO replacement in diets with 16–20% added dietary fat [32,33,34,35]. Nevertheless, when acid oils rich in FFA were used, differences were observed between the two oil sources of different botanical origin. Animals fed SA (53.25% of FFA) achieved a similar performance to that obtained for animals fed crude soybean oil. However, the opposite was obtained for olive pomace acid oil, since animals fed OA (44.95% of FFA) showed the worst performance (lowest SGR and final BW, together with the highest FCR value). In the study by Trullàs et al. [12], where another MUFA-rich acid oil such as rapeseed acid oil was used in rainbow trout diets, no differences were observed in SGR or FCR (42.4–47.3% of FFA) compared to diets containing crude or re-esterified rapeseed oils. A high dietary content of FFA has been associated with lower feed DE values [36,37], which might affect growth. However, in the present study, no differences in digestible energy (DE) of feed among dietary treatments were observed. In addition, a higher MIU value, which estimates the non-energetic fraction of fats and oils, has been associated with a decrease in the DE content of dietary fats [10,38]. The MIU value observed in olive pomace acid oil (6.15%) was 1.7 to 2.5 times higher than those obtained for soybean-sunflower acid oil and the other experimental crude oils, so the MIU content could explain the lower performance observed by fish fed OA. On the other hand, the negative effect of the inclusion of olive pomace acid oil on performance was not observed when this acid oil was blended with soybean oils (crude or acid oil). In this case, a decrease in the MIU content as a result of the blend between oils might contribute to this effect. However, although Trullàs et al. [12] described a lack of negative effects on performance in rainbow trout fed rapeseed acid oil, the MIU values of dietary added fats are not reported in this study. It is therefore important to highlight the need to assess the non-energetic fraction of dietary added fats, especially in the case of acid oils, since as by-products from the edible oil refining industry they can present high variability in their composition depending on the amount and type of compounds removed from crude oil [10].

Both carcass and muscle yields are parameters that may be useful for the industry, as the main valuable final product of aquaculture production is the fillet. However, as far as we know, no studies have assessed the effect of different VO or dietary FFA content on carcass parameters or flesh yield. In the present study, the percentage of FO replacement (75%) with VO (crude or acid, alone or in blends) had no effect on carcass parameters or flesh yield. In addition, lower gross flesh yields were obtained in the present study (40% to 43% of BW) than those reported by Lanari et al. [39] and Vandeputte et al. [40] (44.5% and 57.4% of BW, respectively), which may be related to the smaller size of the animals obtained at the end of the study (226–250 g vs. 316–395 g of BW). On the other hand, similar values of perivisceral fat deposition were obtained for diets including acid oils and their respective crude oils, and also for the other experimental oils or blends, so dietary FFA content and the different compositions of the two added oils of different botanical origin do not seem to be relevant factors in determining perivisceral fat deposition.

### 4.2. Digestibility Balance

The replacement of FO with crude or soybean-sunflower and olive pomace acid oils or blends had no effect on the digestibility of dry matter and crude protein. These results are in agreement with other studies that found no differences in digestibility of nutrients when FO was replaced with different VO [15]. In contrast, the results suggest that the digestibility of lipids decreases when acid oils are included in European seabass diets, as a lower digestibility was obtained in diets containing acid oils (SA, OA and SA-OA) when compared to those containing their corresponding crude oil (alone or in blends). It has been described that increasing the FFA content of the added lipid source has a negative effect on lipid digestibility [36,37] due to the higher melting point and the ability to form insoluble soaps that are unavailable for absorption [41,42]. However, the negative effects of FFA on fish lipid digestibility are controversial in the literature. In contrast to the observations in the present results, Ng et al. [13] described an increase in the ADC values of lipids when replacing FO with palm fatty acid distillates (which are mainly composed of FFA; >90%) in rainbow trout diets. As described above, the higher non-energetic fraction (MIU, especially of unsaponifiable matter) content of the acid oils used in the present study might negatively affect lipid digestibility, leading to a more pronounced decrease in the ADC values of lipids in SA and OA diets [10].

Similar to the observation for ADC of lipids, a decrease in total FA digestibility was observed for the use of acid oil diets, although it was not significant. Trullàs et al. [15] reported a significant decrease in total FA digestibility in rainbow trout fed diets including rapeseed acid oil in comparison to its respective crude oil. In the present study, the lower values for total FA digestibility seem to be related to a lower SFA digestibility in acid oils, as no differences were observed either for MUFA or PUFA. In agreement with this, it is well known that saturated FFA have a greater ability to form insoluble soaps as opposed to unsaturated FFA [43]. In contrast, when diets included acid oils in blends, higher values for ADC of FA were obtained. This effect could be explained by the decrease in the MUFA:PUFA ratio (increasing the level of unsaturation) and/or by the presence of a higher content of other lipid classes such as DAG or MAG, generated from the hydrolysis of TAG (in the case of S-OA), which may enhance the inclusion of FFA from olive pomace acid oil in mixed micelles facilitating their absorption [44,45]. It is important to note that the ADCs of lipids and total FA were high for all dietary treatments (90.7–97.4%), in agreement with results reported by other authors using VO as FO replacers in fish diets [46,47,48].

### 4.3. Flesh Composition and Quality Parameters

In species with a white flesh colour, such as European seabass, preservation of the expected whiteness is a key attribute for determining sensory quality with regard to consumer acceptance [49]. Acid oils can concentrate a higher content of unsaponifiable matter during the refining process of the crude oil that they come from, which consists of many components, including compounds such as sterols, tocopherols, tocotrienols and hydrocarbons, and also pigments that could modify the flesh colour [14]. However, diets including acid oils alone did not show different values to those including crude oils despite the higher content of unsaponifiable matter, especially in the case of olive pomace acid oil, which is characterised by a notable dark colour. In fact, the slight differences in lightness of fresh muscles obtained in the present study did not show a consistent pattern in relation to the dietary treatments. In general, the replacement of crude VO with its acid oils (soybean-sunflower or olive pomace acid oils) did not modify the colour parameters of either fresh or thawed muscle. When comparing fresh to thawed muscle, the present results showed higher L* values in thawed muscles, in agreement with other authors who have confirmed that freezing and storage generally increases flesh brightness [14,50,51].

When the chemical composition and liquid holding capacity of thawed muscle are considered, a non-significant effect of replacing FO with crude and soybean-sunflower and olive pomace acid oils was obtained. These results are in agreement with those found when replacing FO with crude VO such as soybean, rapeseed, linseed and olive oils [34,35,52,53], or with rapeseed acid oil [14]. Similarly, the lipid content of the dorsal and ventral muscle sections was not affected by the botanical origin of the added oil or by FFA content. However, it is important to note that the ventral section of the muscle had approximately twice the amount of lipid content compared to the dorsal section, in agreement with the literature, as it is well known that there is higher fat deposition in the ventral section of the muscle in fish [54,55].

### 4.4. Fatty Acid Profile of Flesh and Perivisceral Fat

The results of the present study suggest that there is no effect of dietary FFA content on the FA profile of flesh and perivisceral fat, but it is affected by the dietary FA profile. The slight differences between diets containing crude or acid oils were those present in the FA profile of the diets. Animals fed S or SA produced flesh that was richer in PUFA and the flesh of those fed O or OA was richer in MUFA. Hence, the inclusion of acid oils in the diets helped to obtain a final product with a similar FA profile to that obtained with animals fed their respective crude oils.

Although the FA composition of flesh and perivisceral fat reflected that of the diet, differences in n − 3 HUFA and C18 FA concentrations were less marked in the FA composition of flesh than that of perivisceral fat. The same effect was observed in other studies performed in European seabass [34,35], in Atlantic salmon (*Salmo salar*) [56,57], in rainbow trout [58] and in gilthead seabream [29]. This could be related to preferential n − 3 HUFA retention in the muscle to maintain an adequate level of fluidity in cell membranes [57,58,59], while the main C18 FA present in VO diets are preferentially used for oxidation processes or are accumulated in the liver [34,56]. Regarding the two sections of flesh, the dorsal section of the muscle showed a higher DHA content than the ventral, which is consistent with the results obtained in European seabass by Campos et al. [60], and could be explained by the higher lipid content in the ventral section of the flesh.

## 5. Conclusions

In conclusion, the substitution of fish oil with different vegetable oils (75%) showed different results depending on the botanical origin and free fatty acid content of the experimental oils. The inclusion of soybean-sunflower acid oil as a replacement for soybean oil does not have a negative effect on performance, feed efficiency or the studied flesh parameters. In contrast, the inclusion of olive pomace acid oil as a replacement for olive pomace oil impaired performance and feed efficiency. However, the negative effects observed for the inclusion of olive pomace acid oil alone disappeared when acid oils were included in a blend with soybean oil or soybean-sunflower acid oil. It is important to note that although similar performance and feed efficiency results could be achieved by including acid oils instead of their respective crude oils, the digestibility of lipids decreased. Hence, the correct evaluation of acid oil quality parameters such as MIU would help to incorporate acid oils in aquaculture diets, since they are highly variable sources in terms of composition.

The present results offer a view on the preliminary step for the potential use of acid oils in farmed fish species. However, further studies assessing the effects of the inclusion of these oils on metabolism, immunology, intestinal health and product quality are needed before recommending their use.

## Figures and Tables

**Table 1:** Ingredients and approximate composition of experimental diets.

Item, g/kg	Experimental Diets							
	F	S	SA	O	OA	S-O	S-OA	SA-OA
Ingredient composition								
Wheat meal	110.34	110.34	110.34	110.34	110.34	110.34	110.34	110.34
Wheat gluten	155.94	155.94	155.94	155.94	155.94	155.94	155.94	155.94
Soya protein concentrate	265.99	265.99	265.99	265.99	265.99	265.99	265.99	265.99
Fish meal	202.45	202.45	202.45	202.45	202.45	202.45	202.45	202.45
Hydrolysed fish protein	25.31	25.31	25.31	25.31	25.31	25.31	25.31	25.31
Krill meal	25.52	25.52	25.52	25.52	25.52	25.52	25.52	25.52
Soybean lecithin	9.62	9.62	9.62	9.62	9.62	9.62	9.62	9.62
Fish oil	153.87	38.47	38.47	38.47	38.47	38.47	38.47	38.47
Experimental oil	-	115.40	115.40	115.40	115.40	115.40	115.40	115.40
L-lysine	2.88	2.88	2.88	2.88	2.88	2.88	2.88	2.88
DL-methionine	0.96	0.96	0.96	0.96	0.96	0.96	0.96	0.96
Choline chloride	4.81	4.81	4.81	4.81	4.81	4.81	4.81	4.81
Betaine	0.96	0.96	0.96	0.96	0.96	0.96	0.96	0.96
Vitamin and mineral premix ^1^	19.23	19.23	19.23	19.23	19.23	19.23	19.23	19.23
Vitamin C	0.96	0.96	0.96	0.96	0.96	0.96	0.96	0.96
Guar gum	19.23	19.23	19.23	19.23	19.23	19.23	19.23	19.23
Yttrium	1.92	1.92	1.92	1.92	1.92	1.92	1.92	1.92
Proximate composition (as-fed basis)								
Dry matter	927.40	928.80	929.70	927.40	928.60	932.50	923.60	926.80
Crude protein	418.30	405.30	396.20	413.10	414.30	419.60	410.90	414.70
Ether extract	190.50	190.40	182.80	186.90	180.00	182.90	186.10	184.20
Ash	72.20	72.40	73.20	72.40	73.40	71.00	71.10	72.10
Gross energy (MJ/kg)	21.72	21.80	21.78	21.69	21.95	21.85	21.75	21.65

Abbreviations: F = fish oil diet (control); S = soybean oil diet; SA = soybean-sunflower acid oil diet; O = olive pomace oil diet; OA = olive pomace acid oil diet; S-O = S and O at 1:1 ratio; S-OA = S and OA at 1:1 ratio; SA-OA = SA and OA at 1:1 ratio. ^1^ Provides, per kg: vitamin A (2,000,000 UI); vitamin D3 (200,000 UI); vitamin E (10,000 mg); vitamin K3 (2500 mg); vitamin B1 (3000 mg); vitamin B2 (3000 mg); calcium pantothenate (10,000 mg); nicotinic acid (20,000 mg); vitamin B6 (2000 mg); vitamin B9 (1500 mg); vitamin B12 (10 mg); vitamin H (300 mg); inositol (50,000 mg); betaine (50,000 mg); cobalt carbonate (65 mg); cupric sulfate (900 mg); iron sulfate (600 mg); potassium iodide (50 mg); manganese oxide (960 mg); sodium selenite (1 mg); zinc sulphate (750 mg); calcium carbonate (186,000 mg); potassium chloride (24,100 mg); sodium chloride (40,000 mg).

**Table 2:** Fatty acid and lipid class composition and MIU values of experimental oils.

Item, %	Experimental Oils				
	FO	SO	SAO	OO	OAO
Fatty acid composition					
SFA	34.93	14.95	16.34	16.84	15.96
MUFA	28.68	25.83	32.33	71.82	66.73
PUFA	36.39	59.22	51.33	11.34	17.31
UFA:SFA	1.86	5.69	5.12	4.94	5.27
Individual fatty acids					
C16:0	21.76	10.73	11.24	13.26	11.54
C18:0	6.41	3.35	3.45	2.73	3.12
C18:1 n − 9	15.96	23.49	30.53	68.65	62.96
C18:2 n − 6	1.88	53.12	48.29	10.43	16.54
C18:3 n − 3	0.89	6.07	3.02	0.85	0.73
C20:5 n − 3	6.32	ND	ND	ND	ND
C22:6 n − 3	26.02	ND	ND	ND	ND
Lipid class composition					
TAG	85.67	93.88	29.31	77.47	36.27
DAG	6.85	4.16	16.10	8.42	17.35
MAG	4.35	0.50	1.34	0.87	1.43
FFA	3.13	1.46	53.25	13.24	44.95
MIU, g/100					
Moisture	0.24	0.05	0.40	0.36	0.31
Impurities	0.30	0.21	0.89	0.44	1.94
Unsaponifiable	2.01	0.53	2.35	1.64	3.90
Total MIU	2.55	0.80	3.64	2.44	6.15

Abbreviations: FO = fish oil; SO = crude soybean oil; SAO = soybean-sunflower acid oil; OO = crude olive pomace oil; OA = olive pomace acid oil; TAG = triacylglycerols; DAG = diacylglycerols; MAG = monoacylglycerols; FFA = free fatty acids; MIU = moisture, impurities and unsaponifiable; ND = non-detected.

**Table 3:** Fatty acid profile of experimental diets.

Item, %	Experimental Diets							
	F	S	SA	O	OA	S-O	S-OA	SA-OA
Fatty acid composition								
SFA	33.11	21.82	22.89	22.03	22.75	22.30	21.86	22.81
MUFA	24.45	24.57	28.49	52.84	48.22	36.26	38.60	38.41
PUFA	42.44	53.61	48.62	25.12	29.03	41.44	39.54	38.78
n − 3	31.55	14.06	12.35	11.01	11.40	12.70	12.43	11.92
n − 6	10.56	39.42	36.14	14.00	17.52	28.60	26.97	26.73
n − 3:n − 6	2.99	0.36	0.34	0.79	0.65	0.44	0.46	0.45
UFA:SFA	1.41	2.18	2.15	2.26	2.28	2.22	2.25	2.20
MUFA:PUFA	0.52	0.68	0.79	1.53	1.42	1.03	1.11	1.09
Individual fatty acids								
C16:0	20.73	14.52	14.96	15.11	15.17	14.85	14.77	15.02
C18:0	6.00	4.24	4.31	3.89	4.11	4.18	4.05	4.22
C18:1 n − 9	15.36	20.19	24.21	47.47	42.34	31.14	33.78	33.33
C18:2 n − 6	8.62	38.85	35.55	13.42	16.92	28.01	26.40	26.13
C18:3 n − 3	1.16	4.61	2.60	1.29	1.24	2.96	3.01	1.94
C20:4 n − 6	1.83	0.57	0.58	0.59	0.60	0.59	0.57	0.60
C20:5 n − 3	6.98	2.47	2.60	2.55	2.72	2.55	2.48	2.63
C22:6 n − 3	23.21	6.97	7.15	7.18	7.44	7.19	6.95	7.36

Abbreviations: F = fish oil diet (control); S = soybean oil diet; SA = soybean-sunflower acid oil diet; O = olive pomace oil diet; OA = olive pomace acid oil diet; S-O = S and O at 1:1 ratio; S-OA = S and OA at 1:1 ratio; SA-OA = SA and OA at 1:1 ratio; SFA = saturated fatty acids; MUFA = monounsaturated fatty acids; PUFA = polyunsaturated fatty acids; UFA = unsaturated fatty acids.

**Table 4:** Performance, feed efficiency and carcass parameters in European seabass fed different dietary fat sources.

Item	Experimental Diets								SEM ^1^	*p*-Value
	F	S	SA	O	OA	S-O	S-OA	SA-OA		
Performance parameters										
BW 0 days (g)	101.29	101.50	101.33	101.36	101.37	101.47	101.34	101.31	0.22	0.997
BW 100 days (g)	250.20 ^a^	245.62 ^a^	244.57 ^a^	247.40 ^a^	226.22 ^b^	244.45 ^a^	244.54 ^a^	246.00 ^a^	3.37	0.002
ADFI (g)	3.34 ^b^	3.57 ^a^	3.42 ^ab^	3.57 ^a^	3.41 ^ab^	3.60 ^a^	3.55 ^ab^	3.59 ^a^	0.05	0.005
ADG (g)	1.49 ^a^	1.44 ^a^	1.43 ^a^	1.46 ^a^	1.25 ^b^	1.43 ^a^	1.43 ^a^	1.45 ^a^	0.03	0.003
FCR	2.246 ^b^	2.480 ^ab^	2.388 ^b^	2.443 ^ab^	2.735 ^a^	2.517 ^ab^	2.480 ^ab^	2.481 ^ab^	0.074	0.004
SGR (%/d)	0.90 ^a^	0.88 ^a^	0.88 ^a^	0.89 ^a^	0.80 ^b^	0.88 ^a^	0.88 ^a^	0.90 ^a^	0.016	0.002
CF	1.94	1.95	1.95	1.99	1.88	2.00	1.93	1.99	0.034	0.206
Carcass parameters										
Carcass weight (g)	219.40	216.33	218.62	215.58	200.19	222.05	225.45	230.43	7.85	0.233
Carcass yield (%)	90.03	90.20	89.12	88.96	89.36	89.14	88.59	88.97	0.53	0.349
Gross flesh yield (%)	42.99	41.96	41.62	41.14	41.47	43.12	40.03	39.66	1.63	0.761
Net flesh yield (%)	47.82	46.70	46.88	46.42	45.16	48.74	45.40	44.56	1.99	0.826
Perivisceral fat (%)	6.22	5.98	6.93	6.76	6.29	7.12	7.10	6.87	0.43	0.341

Abbreviations: F = fish oil diet (control); S = soybean oil diet; SA = soybean-sunflower acid oil diet; O = olive pomace oil diet; OA = olive pomace acid oil diet; S-O = S and O at 1:1 ratio; S-OA = S and OA at 1:1 ratio; SA-OA = SA and OA at 1:1 ratio; BW = body weight; ADFI = average daily feed intake; ADG = average daily gain; FCR = feed conversion ratio; SGR = specific growth rate; CF = condition factor; SEM = standard error of the mean. ^1^ n = 3. ^a,b^ Values within a row with different superscripts differ significantly at *p* < 0.05.

**Table 5:** Feed digestible energy, macronutrient and fatty acid apparent digestibility coefficients in European seabass fed different dietary fat sources.

Item, %	Dietary Treatments								SEM ^1^	*p*-Value
	F	S	SA	O	OA	S-O	S-OA	SA-OA		
DE and macronutrient ADC										
Digestible energy (kcal/kg)	4335.11	4335.05	4234.40	4336.50	4308.66	4485.63	4387.59	4303.19	55.60	0.114
Dry matter	96.34	96.08	95.81	96.02	95.91	96.67	96.21	96.14	0.28	0.388
Crude protein	91.63	89.45	89.16	90.05	89.52	91.96	90.47	90.74	0.89	0.167
Lipids	96.87 ^a^	96.66 ^ab^	93.96 ^cd^	96.26 ^abc^	93.38 ^d^	97.37 ^a^	95.79 ^abc^	94.41 ^bcd^	0.59	<0.001
Fatty acid ADC										
Total fatty acids	90.73 ^c^	93.93 ^ab^	91.77 ^bc^	93.79 ^abc^	91.66 ^bc^	95.56 ^a^	94.26 ^ab^	93.32 ^abc^	0.78	0.003
SFA	79.59 ^cd^	84.37 ^abc^	78.77 ^d^	86.20 ^ab^	81.11 ^bcd^	88.96 ^a^	86.56 ^ab^	83.02 ^abcd^	1.37	<0.001
MUFA	92.31 ^b^	93.92 ^ab^	93.14 ^ab^	95.31 ^ab^	93.65 ^ab^	96.43 ^a^	94.96 ^ab^	95.03 ^ab^	0.83	0.023
PUFA	98.50	97.81	97.09	97.23	96.61	98.37	97.79	97.68	0.52	0.104
n − 3	99.27	98.79	98.39	98.25	98.04	99.15	98.66	98.65	0.36	0.141
n −6	96.16	97.46	96.63	96.40	95.65	98.01	97.39	97.23	0.58	0.072
Individual fatty acids										
C16:0	80.13 ^d^	85.7 ^abc^	81.52 ^cd^	87.48 ^ab^	83.24 ^bcd^	89.81 ^a^	88.05 ^ab^	85.23 ^abcd^	1.31	<0.001
C18:0	75.17 ^cd^	81.04 ^abc^	74.30 ^d^	82.85 ^ab^	76.85 ^bcd^	86.35 ^a^	83.79 ^a^	79.41 ^abcd^	1.66	<0.001
C18:1 n − 9	92.99 ^b^	94.71 ^ab^	93.86 ^ab^	95.71 ^ab^	94.16 ^ab^	96.77 ^a^	95.50 ^ab^	95.58 ^ab^	0.82	0.040
C18:2 n − 6	95.30 ^b^	97.42 ^ab^	96.58 ^ab^	96.24 ^ab^	95.50 ^ab^	97.96 ^a^	97.34 ^ab^	97.17 ^ab^	0.60	0.020
C18:3 n − 3	98.03 ^ab^	98.27 ^ab^	97.33 ^ab^	96.49 ^ab^	95.86 ^b^	98.67 ^a^	98.10 ^ab^	97.55 ^ab^	0.64	0.034
C20:5 n − 3	99.28	99.48	99.16	98.49	98.34	100.00	99.08	99.09	0.54	0.348
C22:6 n − 3	99.33	98.90	98.50	98.48	98.30	99.05	98.75	98.78	0.27	0.091

Abbreviations: DE = digestible energy; ADC = apparent digestibility coefficient; F = fish oil diet (control); S = soybean oil diet; SA = soybean-sunflower acid oil diet; O = olive pomace oil diet; OA = olive pomace acid oil diet; S-O = S and O at 1:1 ratio; S-OA = S and OA at 1:1 ratio; SA-OA = SA and OA at 1:1 ratio; SFA = saturated fatty acids; MUFA = monounsaturated fatty acids; PUFA = polyunsaturated fatty acids; SEM = standard error of the mean. ^1^ n = 3. ^a–d^ Values within a row with different superscripts differ significantly at *p* < 0.05.

**Table 6:** Colorimetric assessment, chemical composition and liquid holding capacity of European seabass flesh according to different dietary treatments.

Colour Parameters ^1^	Dietary Treatments								SEM ^2^	*p*-Value
	F	S	SA	O	OA	S-O	S-OA	SA-OA		
Fresh muscle										
L*	40.21 ^a^	40.24 ^a^	41.63 ^a^	39.62 ^ab^	41.73 ^a^	39.84 ^a^	32.25 ^b^	35.48 ^ab^	1.74	0.001
C*	3.02	3.43	2.99	3.08	3.21	3.55	2.53	2.54	0.26	0.093
h	1.26	1.26	1.25	1.26	1.27	1.21	0.89	1.22	0.10	0.062
a*	0.94	1.22	0.90	0.97	0.99	1.33	0.70	0.90	0.19	0.498
b*	2.82	3.20	2.62	2.87	2.97	3.06	2.37	2.31	0.23	0.058
Thawed muscle										
L*	48.94	47.46	48.61	48.00	49.51	48.16	49.51	47.78	0.77	0.435
C*	4.16 ^a^	3.52 ^ab^	3.68 ^ab^	3.08 ^b^	3.93 ^ab^	2.98 ^b^	3.60 ^ab^	3.25 ^ab^	0.23	0.006
h	−1.30	−1.09	−1.18	−1.04	−1.02	−0.93	−0.84	−0.99	0.11	0.109
a*	−0.98	−1.35	−1.27	−1.48	−1.24	−1.51	−1.52	−1.50	0.13	0.057
b*	3.99 ^a^	3.15 ^abc^	3.39 ^abc^	2.64 ^bc^	3.67 ^ab^	2.39 ^c^	3.08 ^abc^	2.74 ^abc^	0.29	0.002
Chemical composition (%) ^3^										
Moisture	68.23	68.44	69.13	69.56	68.99	68.42	68.02	68.17	0.51	0.393
Organic matter	96.25	96.39	95.99	95.86	95.81	96.25	96.39	95.70	0.39	0.820
Crude protein	63.73	61.47	63.66	65.22	63.56	62.22	62.46	65.05	1.13	0.286
Ash	3.75	3.61	4.01	4.14	4.19	3.75	3.61	4.30	0.39	0.820
Lipid content in dorsal muscle	17.19	17.75	16.93	16.69	16.95	17.04	20.42	16.66	1.95	0.747
Lipid content in ventral muscle	30.12	37.11	35.20	35.48	33.88	38.12	37.93	34.94	2.12	0.235
Liquid holding capacity (as % retained) ^4^										
Drip loss	21.91	23.39	21.68	22.84	23.18	24.83	21.37	23.53	1.10	0.420
Water retained	72.70	72.22	72.27	72.30	76.08	69.73	73.15	70.41	2.02	0.537
Fat retained	86.68	83.80	85.35	84.16	85.67	83.33	88.44	85.11	2.05	0.704

Abbreviations: F = fish oil diet (control); S = soybean oil diet; SA = soybean-sunflower acid oil diet; O = olive pomace oil diet; OA = olive pomace acid oil diet; S-O = S and O at 1:1 ratio; S-OA = S and OA at 1:1 ratio; SA-OA = SA and OA at 1:1 ratio; SEM = standard error of the mean. ^1^ L* = lightness; C* = Chroma = (a*^2^ + b*^2^)^1/2^ (Wyszeki and Stiles, 1967); h = hue = arctan (b*/a*) (Wyszeki and Stiles, 1967); a* = position between red/magenta and green; b* = position between yellow and blue. ^2^ n = 3. ^3^ Values expressed as % of dry matter. ^4^ Measured in thawed muscles. ^a–c^ Values within a row with different superscripts differ significantly at *p* < 0.05.

**Table 7:** Fatty acid composition of dorsal and ventral muscle from European seabass according to different dietary treatments.

Item, %	Dietary Treatments								SEM ^1^	*p*-Value
	F	S	SA	O	OA	S-O	S-OA	SA-OA		
Dorsal muscle										
Fatty acid composition										
SFA	26.46 ^a^	22.38 ^b^	22.61 ^b^	22.40 ^b^	22.49 ^b^	22.46 ^b^	22.33 ^b^	22.36 ^b^	0.16	<0.001
MUFA	32.80 ^c^	32.88 ^c^	34.15 ^c^	48.56 ^a^	46.01 ^a^	39.15 ^b^	40.93 ^b^	40.07 ^b^	0.56	<0.001
PUFA	40.74 ^bc^	44.74 ^a^	43.25 ^ab^	29.04 ^e^	31.51 ^e^	38.39 ^cd^	36.74 ^d^	37.56 ^d^	0.58	<0.001
UFA:SFA	2.78 ^b^	3.47 ^a^	3.42 ^a^	3.46 ^a^	3.45 ^a^	3.45 ^a^	3.48 ^a^	3.47 ^a^	0.03	<0.001
MUFA:PUFA	0.81 ^d^	0.74 ^d^	0.79 ^d^	1.67 ^a^	1.46 ^b^	1.02 ^c^	1.11 ^c^	1.07 ^c^	0.03	<0.001
n − 3	26.93 ^a^	15.84 ^b^	15.82 ^b^	14.72 ^b^	15.05 ^b^	14.96 ^b^	14.57 ^b^	15.47 ^b^	0.44	<0.001
n − 6	12.71 ^d^	27.52 ^a^	26.21 ^a^	13.61 ^d^	15.61 ^c^	22.42 ^b^	21.08 ^b^	21.31 ^b^	0.36	<0.001
n − 3:n − 6	2.13 ^a^	0.58 ^c^	0.60 ^c^	1.08 ^b^	0.96 ^b^	0.67 ^c^	0.69 ^c^	0.73 ^c^	0.04	<0.001
Individual fatty acids										
C16:0	17.82 ^a^	15.30 ^b^	15.52 ^b^	17.73 ^b^	15.66 ^b^	15.42 ^b^	15.56 ^b^	15.44 ^b^	0.14	<0.001
C18:0	4.74 ^a^	4.55 ^ab^	4.52 ^ab^	4.18 ^d^	4.20 ^cd^	4.48 ^abc^	4.22 ^cd^	4.36 ^bcd^	0.06	<0.001
C18:1 n − 9	24.05 ^e^	26.59 ^de^	27.72 ^d^	41.71 ^a^	38.63 ^b^	32.55 ^c^	34.14 ^c^	33.53 ^c^	0.53	<0.001
C18:2 n − 6	11.15 ^d^	26.76 ^a^	25.41 ^a^	12.80 ^d^	14.83 ^c^	21.66 ^b^	20.37 ^b^	20.48 ^b^	0.36	<0.001
C18:3 n − 3	1.85 ^bc^	3.18 ^a^	2.74 ^ab^	1.77 ^c^	1.93 ^bc^	2.69 ^ab^	2.72 ^ab^	2.06 ^bc^	0.19	<0.001
C20:4 n − 6	1.56 ^a^	0.77 ^b^	0.80 ^b^	0.81 ^b^	0.78 ^b^	0.76 ^b^	0.71 ^b^	0.83 ^b^	0.03	<0.001
C20:5 n − 3	5.46 ^a^	3.10 ^bc^	3.21 ^bc^	3.19 ^bc^	3.39 ^b^	3.08 ^c^	3.07 ^c^	3.22 ^bc^	0.06	<0.001
C22:6 n − 3	19.62 ^a^	9.56 ^b^	9.87 ^b^	9.76 ^b^	9.73 ^b^	9.19 ^b^	8.78 ^b^	10.18 ^b^	0.42	<0.001
Ventral muscle										
Fatty acid composition										
SFA	26.00 ^a^	21.97 ^b^	21.99 ^b^	22.04 ^b^	21.86 ^b^	21.86 ^b^	21.90 ^b^	21.74 ^b^	0.21	<0.001
MUFA	35.41 ^c^	33.47 ^c^	35.82 ^c^	50.21 ^a^	48.08 ^a^	40.70 ^b^	42.16 ^b^	41.99 ^b^	0.65	<0.001
PUFA	38.57 ^b^	44.50 ^a^	42.09 ^a^	27.69 ^c^	29.96 ^c^	37.34 ^b^	35.86 ^b^	36.16 ^b^	0.56	<0.001
UFA:SFA	2.85 ^b^	3.55 ^a^	3.54 ^a^	3.53 ^a^	3.57 ^a^	3.57 ^a^	3.56 ^a^	3.60 ^a^	0.04	<0.001
MUFA:PUFA	0.92 ^de^	0.75 ^e^	0.85 ^e^	1.82 ^a^	1.61 ^b^	1.09 ^cd^	1.18 ^c^	1.16 ^c^	0.04	<0.001
n − 3	24.31 ^a^	13.91 ^b^	13.63 ^b^	12.83 ^b^	12.84 ^b^	12.84 ^b^	12.98 ^b^	12.92 ^b^	0.52	<0.001
n − 6	13.13 ^f^	29.12 ^a^	27.21 ^b^	14.03 ^f^	16.18 ^e^	23.41 ^c^	21.77 ^d^	22.18 ^cd^	0.27	<0.001
n − 3:n − 6	1.85 ^a^	0.48 ^d^	0.50 ^d^	0.91 ^b^	0.79 ^bc^	0.55 ^d^	0.60 ^cd^	0.58 ^cd^	0.05	<0.001
Individual fatty acids										
C16:0	17.45 ^a^	15.04 ^b^	15.08 ^b^	15.41 ^b^	15.23 ^b^	15.03 ^b^	15.24 ^b^	15.00 ^b^	0.14	<0.001
C18:0	4.40 ^a^	4.23 ^ab^	4.09 ^bc^	3.83 ^d^	3.82 ^d^	4.08 ^bc^	3.97 ^cd^	3.95 ^cd^	0.05	<0.001
C18:1 n − 9	26.22 ^c^	26.75 ^c^	29.23 ^c^	42.91 ^a^	40.36 ^a^	33.82 ^b^	35.19 ^b^	35.12 ^b^	0.72	<0.001
C18:2 n − 6	11.87 ^g^	28.57 ^a^	26.60 ^b^	13.42 ^f^	15.59 ^e^	22.85 ^c^	21.22 ^d^	21.59 ^cd^	0.28	<0.001
C18:3 n − 3	1.93 ^e^	3.73 ^a^	2.55 ^c^	1.88 ^e^	2.01 ^de^	2.84 ^b^	2.83 ^b^	2.22 ^d^	0.05	<0.001
C20:4 n − 6	1.26 ^a^	0.55 ^b^	0.61 ^b^	0.61 ^b^	0.59 ^b^	0.55 ^b^	0.55 ^b^	0.59 ^b^	0.02	<0.001
C20:5 n − 3	5.15 ^a^	2.86 ^b^	2.98 ^b^	2.96 ^b^	3.10 ^b^	2.77 ^b^	2.84 ^b^	2.90 ^b^	0.10	<0.001
C22:6 n − 3	17.20 ^a^	7.33 ^b^	8.09 ^b^	7.99 ^b^	7.73 ^b^	7.23 ^b^	7.32 ^b^	7.80 ^b^	0.42	<0.001

Abbreviations: F = fish oil diet (control); S = soybean oil diet; SA = soybean-sunflower acid oil diet; O = olive pomace oil diet; OA = olive pomace acid oil diet; S-O = S and O at 1:1 ratio; S-OA = S and OA at 1:1 ratio; SA-OA = SA and OA at 1:1 ratio; SFA = saturated fatty acids; MUFA = monounsaturated fatty acids; PUFA = polyunsaturated fatty acids; UFA = unsaturated fatty acids; SEM = standard error of the mean. ^1^ n = 3. ^a–g^ Values within a row with different superscripts differ significantly at *p* < 0.05.

**Table 8:** Fatty acid composition of perivisceral fat from European seabass according to different dietary fat sources.

Item, %	Dietary Treatments								SEM ^1^	*p*-Value
	F	S	SA	O	OA	S-O	S-OA	SA-OA		
Fatty acid composition										
SFA	26.33 ^a^	21.58 ^b^	22.04 ^b^	21.25 ^b^	21.35 ^b^	21.57 ^b^	21.94 ^b^	21.51 ^b^	0.38	<0.001
MUFA	35.58 ^e^	34.71 ^e^	40.01 ^d^	54.35 ^a^	50.24 ^b^	44.17 ^c^	42.04 ^cd^	44.34 ^c^	0.57	<0.001
PUFA	37.37 ^b^	42.77 ^a^	37.20 ^b^	23.93 ^e^	27.92 ^d^	33.66 ^c^	35.38 ^bc^	33.60 ^c^	0.54	<0.001
UFA:SFA	2.77 ^b^	3.59 ^a^	3.51 ^a^	3.69 ^a^	3.66 ^a^	3.61 ^a^	3.53 ^a^	3.63 ^a^	0.08	<0.001
MUFA:PUFA	0.96 ^ef^	0.81 ^f^	1.08 ^de^	2.27 ^a^	1.80 ^b^	1.32 ^c^	1.19 ^cd^	1.32 ^c^	0.04	<0.001
n − 3	22.99 ^a^	11.06 ^b^	10.68 ^bc^	9.26 ^c^	9.70 ^bc^	10.45 ^bc^	10.28 ^bc^	10.04 ^bc^	0.31	<0.001
n − 6	14.38 ^e^	31.71 ^a^	26.52 ^b^	14.67 ^e^	18.22 ^d^	23.21 ^c^	25.09 ^bc^	23.56 ^c^	0.41	<0.001
n − 3:n − 6	1.60 ^a^	0.35 ^d^	0.40 ^cd^	0.63 ^b^	0.53 ^bc^	0.45 ^cd^	0.41 ^cd^	0.42 ^cd^	0.03	<0.001
Individual fatty acids										
C16:0	17.44 ^a^	14.70 ^b^	15.14 ^b^	14.97 ^b^	14.73 ^b^	15.05 ^b^	15.12 ^b^	14.93 ^b^	0.26	<0.001
C18:0	4.14 ^a^	3.90 ^ab^	3.76 ^ab^	3.33 ^b^	3.43 ^b^	3.64 ^ab^	3.86 ^ab^	3.41 ^b^	0.14	0.009
C18:1 n − 9	25.75 ^e^	28.01 ^e^	32.56 ^d^	46.97 ^a^	42.40 ^b^	37.17 ^c^	35.29 ^c^	37.10 ^c^	0.50	<0.001
C18:2 n − 6	12.99 ^e^	30.99 ^a^	25.77 ^b^	14.04 ^e^	17.54 ^d^	22.55 ^c^	24.43 ^bc^	22.83 ^c^	0.41	<0.001
C18:3 n − 3	2.20 ^cde^	4.05 ^a^	2.75 ^bc^	1.91 ^e^	2.15 ^de^	3.01 ^b^	2.93 ^b^	2.54 ^bcd^	0.11	<0.001
C20:4 n − 6	1.10 ^a^	0.37 ^b^	0.42 ^b^	0.38 ^b^	0.39 ^b^	0.38 ^b^	0.36 ^b^	0.38 ^b^	0.02	<0.001
C20:5 n − 3	5.12 ^a^	2.31 ^b^	2.65 ^b^	2.30 ^b^	2.43 ^b^	2.41 ^b^	2.20 ^b^	2.38 ^b^	0.10	<0.001
C22:6 n − 3	15.48 ^a^	4.59 ^b^	5.17 ^b^	4.96 ^b^	5.02 ^b^	4.93 ^b^	5.05 ^b^	5.01 ^b^	0.35	<0.001

Abbreviations: F = fish oil diet (control); S = soybean oil diet; SA = soybean-sunflower acid oil diet; O = olive pomace oil diet; OA = olive pomace acid oil diet; S-O = S and O at 1:1 ratio; S-OA = S and OA at 1:1 ratio; SA-OA = SA and OA at 1:1 ratio; SFA = saturated fatty acids; MUFA = monounsaturated fatty acids; PUFA = polyunsaturated fatty acids; UFA = unsaturated fatty acids; SEM = standard error of the mean. ^1^ n = 3. Minor fatty acids were considered those that were in <1% proportion. ^a–f^ Values within a row with different superscripts differ significantly at *p* < 0.05.

## Data Availability

Data is available from the corresponding author upon request.
